# Phenotyping for Novel Classification of Adults With Fontan Circulation by Machine Learning

**DOI:** 10.1016/j.jacasi.2026.06.035

**Published:** 2026-08-20

**Authors:** Mitsutaka Nakashima, Norihisa Toh, Tomohisa Seki, Akihito Saito, Katsura Soma, Kodai Saito, Yohei Hyodo, Rie Nakayama, Takashi Miki, Kentaro Ejiri, Hironobu Toda, Yoichi Takaya, Satoshi Akagi, Koji Nakagawa, Nobuhiro Nishii, Toru Miyoshi, Teiji Akagi, Masaru Hatano, Norihiko Takeda, Mark Lachmann, Shinsuke Yuasa

**Affiliations:** a Department of Cardiovascular Medicine, Okayama University Graduate School of Medicine, Dentistry and Pharmaceutical Sciences, Okayama, Japan; b Department of Healthcare Information Management, The University of Tokyo Hospital, Tokyo, Japan; c Department of Cardiovascular Medicine, The University of Tokyo Hospital, Tokyo, Japan; d Department of Cardiovascular Medicine, Niigata University Graduate School of Medical and Dental Sciences, Niigata, Japan; e Department of Cardiovascular Medicine, Hiroshima University Graduate School of Biomedical and Health Sciences, Hiroshima, Japan; f Department of Cardiovascular Medicine and Endocrinology and Metabolism, Faculty of Medicine, Tottori University, Yonago, Japan; g First Department of Medicine, Klinikum rechts der Isar, Technical University of Munich, Munich, Germany

**Keywords:** clustering, decision tree-based analysis, Fontan-type procedure, machine learning

Adults with Fontan circulation have various clinical features, complicating management. We aimed to clarify potential phenotypes and develop a clinically applicable guide using machine learning.

This was a multicenter retrospective cohort study, with derivation set (Okayama University Hospital, 2004-2023) and validation set (the University of Tokyo Hospital, 2009-2024). We enrolled consecutive adults (≥18 years) with Fontan circulation, excluding those lacking cardiac catheterization or laboratory tests. The study protocol involved: 1) agglomerative hierarchical clustering in derivation set using baseline characteristics; 2) constructing a decision tree predicting these clusters in derivation set; and 3) external validation. Ethics committees of Okayama University Hospital (2409-004) and the University of Tokyo Hospital (2024453NIe) approved. Informed consent was waived.

Continuous variables with missing value ratio <0.3 in both derivation set and validation set were analyzed: general characteristics (age at entry [Age], age at Fontan procedure and interval since Fontan procedure), cardiac catheterization (systolic arterial pressure, Fontan pressure [FP],^1^ pulmonary artery wedge pressure [PAWP], cardiac index and arterial oxygen saturation) and laboratory data (white blood cell count, hemoglobin, platelet count, serum albumin [Alb], total bilirubin, aspartate aminotransferase [AST], alanine aminotransferase, creatinine, and brain natriuretic peptide). Variables were selected based on principal component analysis.^2^^,^^3^ Data were standardized, outliers were excluded and missing values were imputed using random forest-based single imputation method (“missForest” package). Distributions of imputed values were compared using density plots. The ideal cluster number was determined using Elbow method based on the within-clusters sum of squares. Euclidean distance metric and Ward's minimum variance method were calculated. We constructed a decision tree predicting the clusters using recursive partitioning analysis by 5-fold cross-validation. Prediction performance was evaluated by micro- and macro-F1. In validation set, reference clusters were determined as the cluster with the shortest Euclidean distance to the centroid of each cluster obtained from clustering in the derivation set. Event-free rates of clinical endpoints after enrollment were evaluated in log-rank tests, and Cox proportional hazards modelling was used to estimate HRs and 95% CIs. Clinical endpoints were defined as composite outcomes of all-cause deaths and heart failure hospitalizations (HHFs). The clinical endpoints were reviewed before machine learning analysis. The Holm method was used in pairwise testing. These analyses were performed using the R statistical package (version 4.4.0; R Foundation for Statistical Computing).

After excluding 13 patients, the derivation set contained 104 patients (25 ± 9 years, 53.8% men [n = 56/104]). We identified 3 clusters based on 6 variables: age, interval since Fontan procedure, FP, PAWP, Alb, and AST: missing values were found in FP (8.7% [n = 9 of 104]), and PAWP (9.6% [n = 10 of 104]) (Figure 1A).

**Figure 1:**
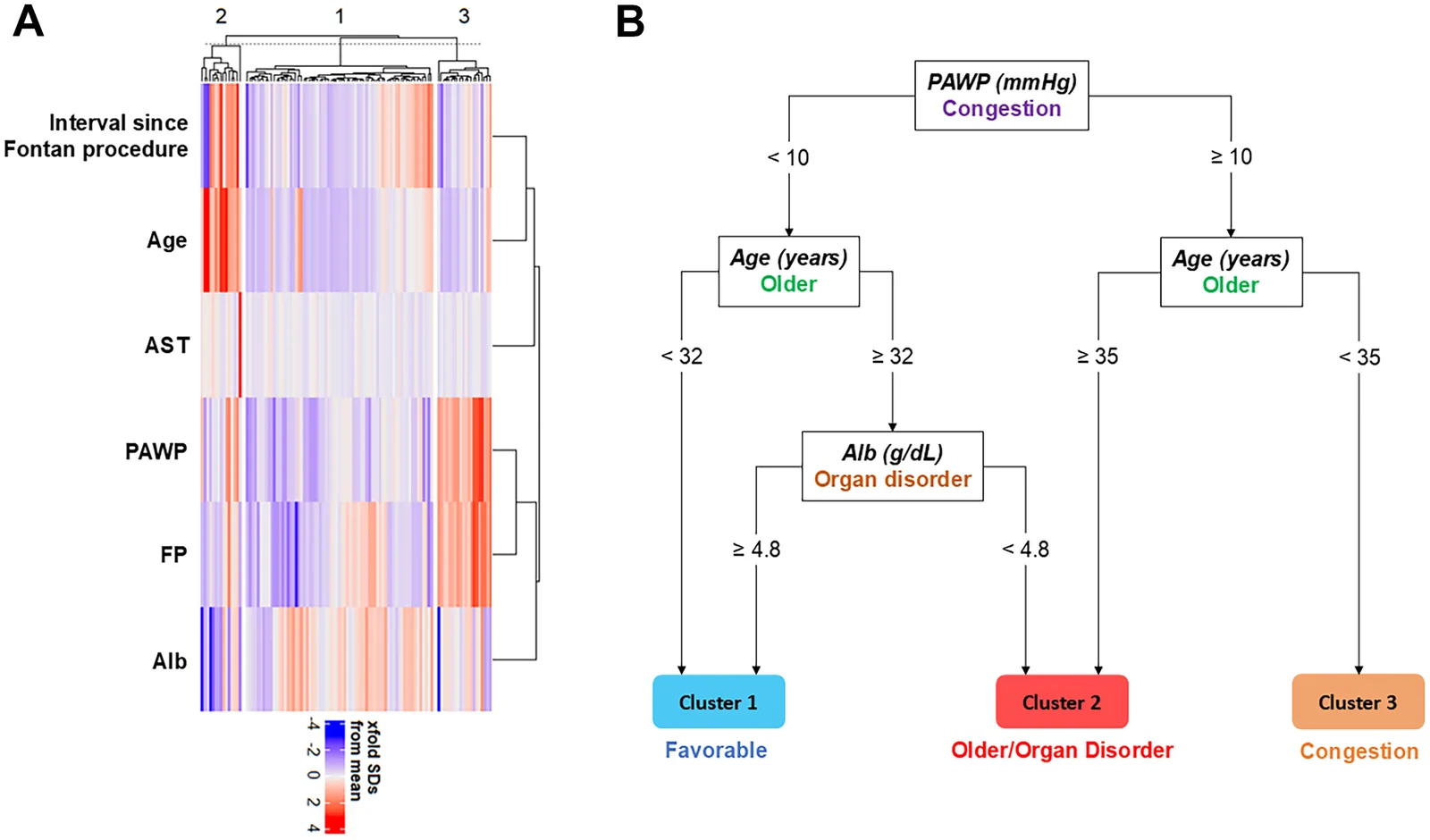
Results of Machine Learning Analysis (A) Heatmap and dendrogram for visualization of clustering revealing 3 phenotypes in adult patients with Fontan circulation: cluster 1 “Favorable,” cluster 2 “Older/Organ Disorder,” and cluster 3 “Congestion.” (B) A simple decision tree predicting clusters constructed by PAWP, Age, and Alb. Age = age at entry; Alb = serum albumin; AST = aspartate aminotransferase; FP = Fontan pressure; PAWP = pulmonary artery wedge pressure.

Cluster 1 (n = 69, “Favorable”): preserved Alb, lowest PAWP.

Cluster 2 (n = 15, “Older/Organ disorder”): oldest, worst AST and Alb.

Cluster 3 (n = 20, “Congestion”): highest FP and PAWP (16 ± 2 and 12 ± 2 mm Hg).

During median, 4.7 [3.4-6.9] years, 10 clinical endpoints occurred (all-cause death, n = 2 [sudden death and malignant mesothelioma]; HHF, n = 8) in the derivation set. Clinical endpoints occurred 1.4% (n = 1 of 69) in cluster 1, 40.0% (n = 6 of 15) in cluster 2, and 15.0% (n = 3 of 20) in cluster 3 (log-rank test, *P* = 0.02). Cluster 1 had better prognosis than cluster 2 (pairwise testing, *P* = 0.019). The 4-year survival rate was 98.6% (95% CI: 90.2%-100.0%) in cluster 1, 91.7% (95% CI: 53.9%-98.8%) in cluster 2, and 94.1% (95% CI: 65.0%-99.1%) in cluster 3. The HRs referencing cluster 1 were 12.6 (95% CI: 1.4-112.5) in cluster 2 and 8.6 (95% CI: 0.9-84.1) in cluster 3.

A decision tree was constructed (Figure 1B). Micro- and macro-F1 performances of this decision tree were 0.962 (95% CI: 0.923-0.99) and 0.942 (95% CI: 0.862-0.99) in the derivation set.

After excluding 3 patients, the validation set contained 106 patients (23 ± 6 years, 60.4% men [n = 64 of 106]). Characteristics of clusters by decision tree in the validation set were consistent with the derivation set (cluster 1 [n = 75]: lower PAWP; cluster 2 [n = 11]: oldest; cluster 3 [n = 20]: highest FP and PAWP). During median, 3.5 (2.0-5.9) years, 14 clinical endpoints occurred (all-cause death, n = 5 [sudden cardiac death, sepsis and bleeding event, and 2 patients died by unknown cause]; HHF, n = 9). Clinical endpoints occurred in 6.7% (n = 5 of 75) in cluster 1, 45.5% (n = 5 of 11) in cluster 2 and 20.0% (n = 4 of 20) in cluster 3, log-rank test, *P* = 0.003. Cluster 1 had a better prognosis than cluster 2 (*P* = 0.005) and cluster 3 (*P* = 0.012) in pairwise testing. The 4-year survival rate was 98.4% (95% CI: 88.9%-99.8%) in cluster 1, 49.1% (95% CI: 12.3%-78.5%) in cluster 2, and 77.5% (95% CI: 50.4%-91.0%) in cluster 3. The HRs referencing cluster 1 were 6.0 (95% CI: 1.7-21.1) in cluster 2 and 2.4 (95% CI: 1.3-4.6) in cluster 3. Micro- and macro-F1 performances of the decision tree were 0.698 (95% CI: 0.613-0.783) and 0.548 (95% CI: 0.455-0.629) to predict clusters in the validation set.

Although cluster 1 showed favorable parameters and prognosis, clinical status may deteriorate over time, necessitating continuous follow-up. Cluster 2 showed older and worse Alb and liver disorder, and cluster 3 was characterized by elevated FP and PAWP. These findings are established as prognostic factors and need careful management.^4^, ^5^, ^6^, ^7^, ^8^, ^9^, ^10^ The decision tree used the cutoff of PCWP ≥10 mm Hg, whereas >12 mm Hg was reported to be associated with prognosis,^9^ suggesting that lower PAWP may be appropriated to distinguish phenotypes. The cutoff of Age in the decision tree was consistent with that in a previous study reported as the risk of worse prognosis.^10^ Although FP, interval since Fontan procedure, and AST were not included in the final decision tree, their effects may have been captured by PAWP, Age, and Alb, which better reflected overall clinical status. Cutoff value of Alb 4.8 g/dL in the decision tree is higher than the lower limit of normal range. Because various factors contribute to hypoalbuminemia, those who maintain a relatively high Alb may represent better overall systemic conditions. On the other hand, AST may not provide sufficient additional discriminatory value after the splits by PAWP, Age, and Alb.

This study had several limitations. First, this study had a small sample size, especially clinical endpoints. Second, some variables had missing values, although the missing value ratio was <0.3 according to previous reports, which may still introduce uncertainty.^2^^,^^3^ Third, echocardiographic, cardiopulmonary exercise testing, and clinical history data were excluded because of the difficulty of standardized assessments, high missing value ratio or noncontinuous formats. Finally, we used only continuous variables in machine learning.

Machine learning appropriately stratified adults with Fontan circulation into phenotypes with different clinical outcomes and predicted them using a simple decision tree model.

## Funding Support and Author Disclosures

This study was supported by the Japanese Circulation Society (Tokyo, Japan) for the 2024 Promotion of Clinical Research Grant. This work has no relationship with industry. The authors have reported that they have no relationships relevant to the contents of this paper to disclose.
